# Mismatch negativity and P3a in drug-naive adults with attention-deficit hyperactivity disorder

**DOI:** 10.1017/S0033291720005516

**Published:** 2022-11

**Authors:** Ming H. Hsieh, Yi-Ling Chien, Susan Shur-Fen Gau

**Affiliations:** 1 Department of Psychiatry, National Taiwan University Hospital and College of Medicine, Taipei, Taiwan; 2 Graduate Institute of Brain and Mind Sciences, and Graduate Institute of Clinical Medicine, College of Medicine, National Taiwan University, Taipei, Taiwan

**Keywords:** Adult ADHD, drug-naive, mismatch negativity, neurophysiological marker, P3a

## Abstract

**Background:**

Individuals with attention-deficit hyperactivity disorder (ADHD) often display over-response to stimuli that are irrelevant to the ongoing task, and their attentional abilities disproportionately worsen in the presence of competing stimuli. Auditory event-related potentials (ERPs) such as mismatch negativity (MMN) and P3a using the passive oddball paradigm have been studied in children and adolescents with ADHD. Still, there is no such data for adults with ADHD. This study aimed to compare the MMN and P3a and their clinical and neurocognitive correlations between drug-naive adults with ADHD and control adults.

**Methods:**

We recruited 52 adults with ADHD (26.5 ± 6.2 years), and 62 age-matched controls (25.6 ± 5.6 years). They received the psychiatric interviews, auditory ERP, the Conners' continuous performance test (CCPT), and the Cambridge gambling test (CGT). They also completed the questionnaires about ADHD symptoms and real-world executive functions. MMN and P3a were assessed during a passive duration-deviant auditory oddball paradigm from the midline electrodes Cz.

**Results:**

Adults with ADHD demonstrated smaller Cz MMN amplitude, more severe ADHD symptoms, poorer attention profiles (CCPT), and a wide range of executive dysfunctions than controls. As for the correlates, Cz peak amplitude of MMN correlated with inattention symptoms, executive dysfunctions, attentional vigilance (CCPT), and decision-making (CGT) in ADHD adults but only with decision-making in controls.

**Conclusions:**

Our findings that smaller amplitude of MMN and its differential associated pattern with inattention, real-world executive dysfunction, and decision-making, in drug-naive adults with ADHD from adult controls, provide evidence to support the potential electrophysiological biomarker for adult ADHD.

## Introduction

Attention-deficit hyperactivity disorder (ADHD) is a prevalent neurodevelopmental disorder that affects 11% of children in the USA (Centers for Disease Control and Prevention, 2016) and 7–9% in Taiwan (Chen, Chen, Lin, Shen, & Gau, 2019; Gau, Chong, Chen, & Cheng, 2005). Beyond clinical symptoms, executive function deficits constitute another hallmark of ADHD (Barkley, 1997; Barkley & Murphy, 2010; Biederman *et al*. 2006; Biederman *et al*. 2007; Faraone *et al*. 2006; Pennington & Ozonoff, 1996) and are hypothesized to be responsible for some clinical symptoms like inattention symptoms and risky behaviors lasting to adulthood (Groen, Gaastra, Lewis-Evans, & Tucha, 2013 Shoham, Sonuga-Barke, Yaniv, & Pollak, 2019). Executive functions are defined as a group of high-order cognitive functions necessary for goal-directed activities, of which working memory (WM) and inhibition are prominent components (Biederman *et al*. 2007; Karch *et al*. 2010; Tseng & Gau, 2013). WM and its predecessor, freedom from distractibility, refer to the ability to hold information in mind for complex tasks (Alderson, Kasper, Hudec, & Patros, 2013; Baddeley, 1998). At the same time, inhibition deficiency underpins risky behaviors or impaired impulse control in ADHD (Barkley, 1997; Pennington & Ozonoff, 1996). Searching for the underlying brain function involved in these long-lasting neuropsychological impairments is of particular interest.

The auditory event-related potential (ERP) was used to assess the brain's electrical activity in response to the auditory stimulation (Escera, Alho, Schroger, & Winkler, 2000). Among the auditory ERP paradigms, mismatch negativity (MMN) and P3a involve preattentive change detection and involuntary orientation to changes in a sequence of otherwise repetitive stimuli, which subjects do not need to pay attention to (Escera *et al*. 2000). MMN, which is generated when a discernible change occurs in a series of repetitive standard stimuli (Naatanen, Gaillard, & Mantysalo, 1978), represents the preattentive process of novelty detection and is associated with auditory memory and involuntary attention shifting (Javitt, Doneshka, Grochowski, & Ritter, 1995; Naatanen & Michie, 1979; Naatanen, Paavilainen, Rinne, & Alho, 2007). P3a is the positive deflection automatically arising after the MMN waveform and has a frontal/central maximum amplitude distribution. In contrast to MMN, which is attention-independent, P3a is an index for the switch of attention (Naatanen *et al*. 2007; Yang *et al*. 2015).

ADHD patients often display over-response to stimuli that are irrelevant to the ongoing task, and their attentional abilities disproportionately worsen in the presence of competing stimuli (Gumenyuk *et al*. 2005). Therefore, previous studies utilized MMN as a preattentional, automatic biomarker to evaluate ADHD patients (Earls, Curran, & Mittal, 2016; Gomes, Duff, Flores, & Halperin, 2013; Oades, Dittmann-Balcar, Schepker, Eggers, & Zerbin, 1996; Rothenberger *et al*. 2000; Sawada *et al*. 2010; Winsberg, Javitt, Silipo, & Doneshka, 1993). A meta-analysis of six MMN studies by Cheng *et al*. (2016), suggested that children with ADHD demonstrated reduced MMN amplitude compared with healthy children. However, these studies are limited by a very small sample size (*<*15 for each group), restricted on children and adolescents, possible medication effect as MMN reported to be influenced by methylphenidate (Winsberg *et al*. 1993) and inconsistent MMN parameters (e.g. frequency MMN, duration MMN, and speech sounds MMN). Hence, a comprehensive study of a larger sample of drug-naive individuals with ADHD and controls beyond child samples is highly indicated.

There have been few studies measuring P3a in ADHD patients, and some of them collected P3a during a distracting task (Gumenyuk *et al*. 2005; Liotti, Pliszka, Perez, Kothmann, & Woldorff, 2005; Oja *et al*. 2016; van Mourik, Oosterlaan, Heslenfeld, Konig, & Sergeant, 2007; Wild-Wall, Oades, Schmidt-Wessels, Christiansen, & Falkenstein, 2009). In contrast, only two studies collected P3a via passive auditory change detection. Applying both pure tones and lexical tones deviations in 15 children with ADHD and 16 age-matched controls, 6–15 years of age, Yang *et al*. (2015) found attenuated P3a in children with ADHD without group difference in MMN. Rydkjaer *et al*. (2017), recorded MMN and P3a using passive four-tone auditory oddball task (standard, frequency deviants, duration deviants, and frequency/duration deviants), found that ADHD adolescents (*N* = 28) showed marginally smaller MMN amplitudes for midline electrodes than controls without significant group differences in P3a amplitude for midline electrodes (Fz/FCz/Cz).

Whether ERP abnormalities noted in children with ADHD persist into their adulthood waits to be tested. Previous adult ADHD studies only utilized go/no-go paradigm, which is task-dependent (Kakuszi, Tombor, Papp, Bitter, & Czobor, 2016; Karch *et al*. 2010). On the contrary, as MMN and P3a under passive auditory paradigm require no overt behavioral response and can be elicited even in the absence of directed attention (Gau, Tseng, Tseng, Wu, & Lo, 2015; Shang & Gau, 2012; Tseng & Gau, 2013), it is interesting to explore the relationships between these electrophysiological markers and ADHD core symptoms, executive dysfunctions, and inhibition deficits. A meta-analytic review of 38 adult ADHD studies indicates WM deficits in ADHD persist into adulthood (Alderson *et al*. 2013). In contrast, a systematic literature review reported that evidence for increased risky performance, assessed by gambling tasks, in ADHD, is mixed but is stronger for children/adolescents with ADHD than for adults with ADHD, and several factors may increase the possibility for risk-taking behaviors in ADHD (Groen *et al*. 2013). For instance, comorbid intellectual disability, ADHD subtypes, methylphenidate use, and the form of reward received may affect risky performance in gambling tasks. Interestingly, whether novelty detection (MMN) and reorientation brain responses (P3a), measured by ERP, influence their performance on gambling tasks or not is unclear.

The current study is the first study to clarify ERP responses by testing both MMN and P3a in drug-naive adults with ADHD and to examine how these electrophysiological markers relate to behavioral and neuropsychological phenotypes of attention and executive functions. Our first hypothesis is that, as found in children with ADHD (22), adults with ADHD may still have reduced MMN amplitude compared to healthy adult controls, which are correlated with inattention and hyperactivity-impulsivity of ADHD core symptoms. Second, we hypothesize that the brain responses towards novelty detection and attention reorientation measured by MMN and P3a can predict the real-life and neuropsychologically assessed attention deficits and executive dysfunctions in adults with ADHD.

## Methods

This study was approved by the Research Ethics Committee of National Taiwan University Hospital, Taipei, Taiwan (Approval number, 201401024RINC; ClinicalTrials.gov number, NCT02642068). All the participants provided written informed consent after a detailed explanation of the procedures and purpose of the study.

### Participants and procedures

We recruited 52 drug-naive adults with the persistent clinical diagnosis of ADHD [36 men, 69.2%; mean age ± standard deviation (s.d.): 26.5 ± 6.2 years] conducted by the corresponding author for childhood ADHD according to the Diagnostic and Statistical Manual of Mental Disorders, Fourth Edition (DSM-IV) diagnostic criteria (American Psychiatric Association, 1994) and also the current ADHD diagnosis according to the Diagnostic and Statistical Manual of Mental Disorders, Fifth Edition (DSM-5) criteria (American Psychiatric Association, 2013) at the outpatient psychiatric clinics of the National Taiwan University Hospital, Taipei, Taiwan. We also recruited 62 healthy adult controls without ADHD (39 men, 62.9%; mean age ± s.d.: 25.6 ± 5.6 years) from the same school via teachers' referrals or the same community via advertisement according to the age and sex distribution of the adult ADHD group. All the participants received clinical evaluation and psychiatric interviews with modified ADHD supplement (Lin & Gau, 2019, 2020; Lin, Yang, & Gau, 2016) of the *Chinese Kiddie epidemiologic version of the Schedule for Affective Disorders and Schizophrenia* (*K-SADS-E*) interview (Gau *et al*. 2005) and the Chinese version of the *Modified Schedule of Affective Disorder and Schizophrenia-Lifetime* (*SADS-L*) (Endicott & Spitzer, 1978; Lin *et al*. 2016; Lin & Gau, 2019; 2020; Merikangas *et al*. 1998; Ni *et al*. 2013; Wu *et al*. 2011), a semi-structured interview based on the Diagnostic and Statistical Manual of Mental Disorders, 4th Edition, Text Revision (DSM-IV-TR) criteria for the diagnosis of ADHD and to exclude other major neuropsychiatric disorders like autism spectrum disorder, schizophrenia, mood disorders, anxiety disorders, substance use disorder, or neurological disorders. Additionally, adults with a full-scale intelligence quotient (IQ) score, assessed by the Wechsler Adult Intelligence Scale-third edition, <80 years of age were also excluded from the study. All the participants performed the Conners' continuous performance test for windows II (CCPT), the Cambridge neuropsychological test automated battery (CANTAB), and ERP with a passive auditory oddball paradigm, as well as completed the questionnaires.

### Behavioral Measures (see Supplementary Methods)

***The Chinese version of the Swanson, Nolan, and Pelham, version IV scale (SNAP-IV)*.** The participants reported their ADHD-related symptoms on the first 18 items of SNAP-IV (Swanson *et al*. 2001; Yang, Tai, Yang, & Gau, 2013), parallel to the core symptoms of DSM-IV ADHD (items 1–9 for inattention symptoms; items 10–18 for hyperactivity/impulsivity symptoms) (see online Supplementary methods). Items are rated on a 4-point Likert scale (0 for ‘not at all’ to 3 for ‘very much’). The psychometric properties of the Chinese SNAP IV have been established (Gau *et al*. 2008; Gau *et al*. 2009).

***The Behavior Rating Inventory of Executive Function (BRIEF)***. The BRIEF (Gioia, Isquith, Guy, & Kenworthy, 2000), an 86-item questionnaire, is designed to be reported by adults aged 18 and older about their real-world executive functions (Baron, 2000). Eight clinical subscales are collapsed into two broad indices: (1) *behavioral regulation index (BRI)*: inhibit, shift and emotional control and (2) *metacognition index (MCI)*: initiate, WM, planning and organizing, organization of materials and monitor; as well as an overall index (Global Executive Composite). Items are rated as 1 (never), 2 (sometimes), and 3 (often). The BRIEF in Chinese has been used in epidemiological (Tsai, Chen, & Gau, 2019) and clinical (Goto *et al*. 2017) research.

### Neurocognitive tasks (see Supplementary Methods)

#### Conners' continuous performance test

The CCPT (see online Supplementary methods) is a widely used computerized task to assess attention performance by non-X type CPT test of go/no-go paradigm (Conners & Staff, 2000). The 360 trials, composed of 10% no-go targets, were presented with six blocks and three sub-blocks (20 trials in each sub-block) with randomly organized sequences of inter-stimulus intervals (ISIs) as 1, 2, and 4 s. Seven indexes represent the three attention profiles (Egeland & Kovalik-Gran, 2010) namely: (1) *focused attention*: omission errors, reaction time (RT) variability, and Hit RT standard errors (s.e.); (2) *cognitive impulsivity*: commission errors and perseverations; and (3) *vigilance*: Hit RT and Hit RT s.e. changed across different ISIs.

#### Cambridge gambling test

Cambridge gambling test (CGT), one of the tasks of the CANTAB (Cambridge Cognition Ltd) tasks, is designed to assess decision-making ability (Rogers *et al*. 1999), while the participant is presented with ten boxes, colored either red or blue and appeared in varying ratios (6:4, 7:3, 8:2, and 9:1) of red to blue. They are informed that a yellow token is hidden in one of the boxes. At the bottom of the screen are two response boxes, one for each color. The participant must use such information to guess whether the token is hidden under a red or blue box. If the participant had located the hidden token correctly, then the points they wagered were added to their total score. If they had made the wrong decision, however, then the same amount was subtracted from their total. These bet amounts were presented either in ascending or descending order during CGT administration. Participants were required to choose a wager from any of these possible amounts within 2 s. If they failed to do so, then the last bet was automatically set by the computer. Their bets were presented together with a sound (low-pitched tones, low bets; high-pitched tones, and high bets). The detailed experimental procedure is presented in the online Supplementary Methods. Six indexes are presented: (1) *Overall bet proportion*: both the ascending and the descending conditions, (2) *risk adjustment*: the mean risk-taking score (points) for each box ratio for both conditions where points to gamble differ relative to box ratio, and (3) *Risk-taking*: the total difference between risk-taking scores (points gambled) in both the ascending and descending conditions (delay aversion).

### ERP recording and processing

MMN and P3a were collected and processed following the standard protocols (Duncan *et al*. 2009; Light *et al*. 2010) and had been used in our previous research (Hsieh *et al*. 2012; Hsieh *et al*. 2019; Huang *et al*. 2018; Lin *et al*. 2012; Lin *et al*. 2014) (details in online Supplementary Methods). In brief, subjects were seated in a comfortable recliner and instructed to relax with his/her eyes open and to focus on the video monitor watching a silent cartoon in a sound-attenuated and electrically shielded booth during the passive auditory oddball paradigm. The stimuli were generated and data was recorded by Neuroscan STIM and ACQUIRE systems. Electrodes placed at the tip of the nose and Fpz served as the reference and ground, respectively. Auditory stimuli were presented binaurally via foam insert headphones using a duration-deviant auditory oddball paradigm, in which standard (*p* = 0.90, 50-ms duration) and deviant (*p* = 0.10, 100-ms duration) tones were presented in a pseudorandom order with at least 2 standards are presented before each deviant. All stimuli were 1000 Hz and 80 dB with 1 ms rise–fall time and presented at a fixed 500 ms onset-to-onset asynchrony. Electroencephalographic (EEG) acquisition was terminated when a minimum of 225 artifact-free deviant trials were collected, while the whole session took over ~30 min in duration.

Offline data processing was performed with automated procedures utilizing Neuroscan Scan 4.5 software blind to the clinical group, and the continuous data files were epoched 100 ms prestimulus to 500 ms post-stimulus. MMN and P3a indices were the peak amplitude/latency between 90 and 250 ms, 210 and 350 ms from the midline electrodes Cz, respectively (Duncan *et al*. 2009; Rydkjaer *et al*. 2017). The measure of global field power (GFP), defined as the s.d. across multiple channels as a function of time and constituted by a single, reference-independent measure of response strength considering the data from all recording electrodes simultaneously, were computed on stimuli and difference waveforms (Skrandies, 1990). Peaks of GFP reflect a maximum of the total underlying brain activity that contributes to the surface potential field (i.e. MMN and P3a) (Shimano *et al*. 2014; Takahashi *et al*. 2013). Rain cloud plots with boxplots were also demonstrated for data visualization (Allen, Poggiali, Whitaker, Marshall, & Kievit, 2019).

### Statistical analysis

Data was analyzed using SAS version 9.4 (SAS Institute Inc., Cary NC, USA). To compare the variables between the ADHD and control groups Student's *t* tests for continuous variables and χ^2^ tests for categorical variables were used. For MMN and P3a parameters, distributions were tested for normality using the Shapiro–Wilk test with a significance level set at *p* value of 0.01 level, and would use a non-parametric Mann–Whitney test when the distributions differ from normality. Pearson correlations analysis was used to correlate the index(es) from MMN/P3a if showing significant group differences, and clinical and neuropsychological measures. All tests were done as two-tailed tests with an *α*-level of *p* *<* 0.05. To control for the inflation of Type I error in computing multiple bivariate correlations, multiple linear regression models with the backward elimination procedure were conducted to determine the relationship between MMN (Cz) amplitude, treated as a dependent variable, and the clinical and neuropsychological measures, treated as independent variables. In order to investigate disease-specific patterns in adults with ADHD, which was hypothesized to be different from the patterns in adult controls, we conducted the above-mentioned multiple regression analyses stratified by the ADHD and control groups. To validate the results of the backward elimination procedure, we also performed forward and stepwise selection, as well as the least absolute shrinkage and selection operator (Lasso) for model selection to identify the variables which were consistently associated with MMN or P3a parameters in the ADHD group. Lasso is a regression analysis method that performs both variable selection and regularization in order to enhance the prediction accuracy and interpretability of the statistical model (Tibshirani, 1996).

## Results

Demographics, ADHD symptom profiles, and MMN/P3a parameters are demonstrated in Table 1. There were no differences regarding age (ADHD: 26.5 ± 6.2 years of age and control: 25.6 ± 5.6years of age), gender, education, employment status, and IQ profiles. Regarding clinical symptoms measured by the SNAP-IV, ADHD adults had significantly more severe inattentive and hyperactive/impulsive symptoms than controls.

**Table 1.** Demographics, attention-deficit hyperactivity disorder (ADHD) symptom profiles, MMN/P3a parameters for adults with ADHD and controls

Mean ± s.d.	ADHD (*N* = 52)	Controls (*N* = 62)	*F* or χ^2^	*p*	
Age (in years)	26.47 ± 6.15	25.61 ± 5.60			
Gender: male, *N*(%)	36 (69.23)	39 (62.90)	0.50	0.478	
Educational level, *N*(%)			3.82	0.051	
College or higher	40 (76.92)	56 (90.32)			
Senior high school	12 (23.08)	6 (9.68)			
Employment status, *N*(%)			0.96	0.328	
Skilled work	24 (46.15)	23 (37.10)			
Others	28 (53.85)	39(62.90)			
Full-scale IQ	106.42 ± 10.75	107.21 ± 9.56	0.17	0.680	
Verbal IQ	104.21 ± 11.20	106.03 ± 9.51	0.88	0.350	
Performance IQ	108.81 ± 10.84	107.90 ± 11.08	0.29	0.662	
SNAP-IV					
Inattentive (0–27)	19.34 ± 5.00	7.23 ± 4.54	183.18	*<*0.001	
Hyperactive/impulsive (0–18)	14.35 ± 6.34	3.71 ± 3.98	118.87	*<*0.001	
MMN					Effect size (Cohen's *d*)
Cz latency	153.37 ± 25.32	159.48 ± 23.60	1.78	0.185	0.250
Cz amplitude	−1.75 ± 0.97	−2.18 ± 1.04	5.33	0.023	0.436
Cz amplitude^a^				0.007	0.295
P3a					
Cz latency	260.17 ± 15.39	256.37 ± 12.21	2.16	0.144	0.274
Cz amplitude	3.99 ± 1.99	4.69 ± 2.51	2.63	0.108	0.308
Cz amplitude^b^				0.129	0.166

Abbreviations: ADHD, attention-deficit hyperactivity disorder; s.d., standard deviation; SNAP-IV, The Chinese version of the Swanson, Nolan, and Pelham, version IV scale.

a Shapiro–Wilk test showed deviation from normality in MMN Cz amplitude of ADHD (*p* = 0.005) and controls (*p* = 0.002). Therefore, non-parametric Mann–Whitney test was used and effect sizes were calculated by the rank biserial correlation.

b Shapiro–Wilk test showed deviation from normality in P3a Cz amplitude of ADHD (*p* = 0.013) and controls (*p* *<* 0.001). Therefore, non-parametric Mann–Whitney test was also used and effect sizes were calculated by the rank biserial correlation.

**Fig. 1.**
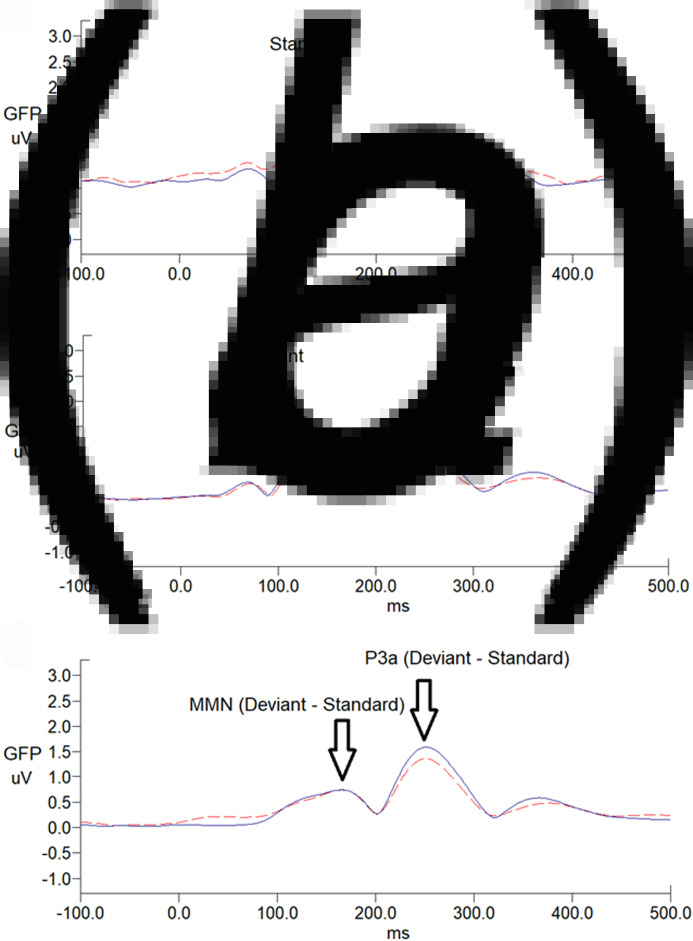
Grand average global field power waveforms of responses to (*a*) standard stimuli, (*b*) deviant stimuli, and (*c*) MMN waveforms, followed by P3a in adults with ADHD (*n* = 52; dash line) and age-matched controls (*n* = 62; solid line). MMN/P3a difference waveforms were obtained by subtracting ERP waveforms elicited by the standard stimuli (a) from those of the deviant stimuli (b).

**Fig. 2.**
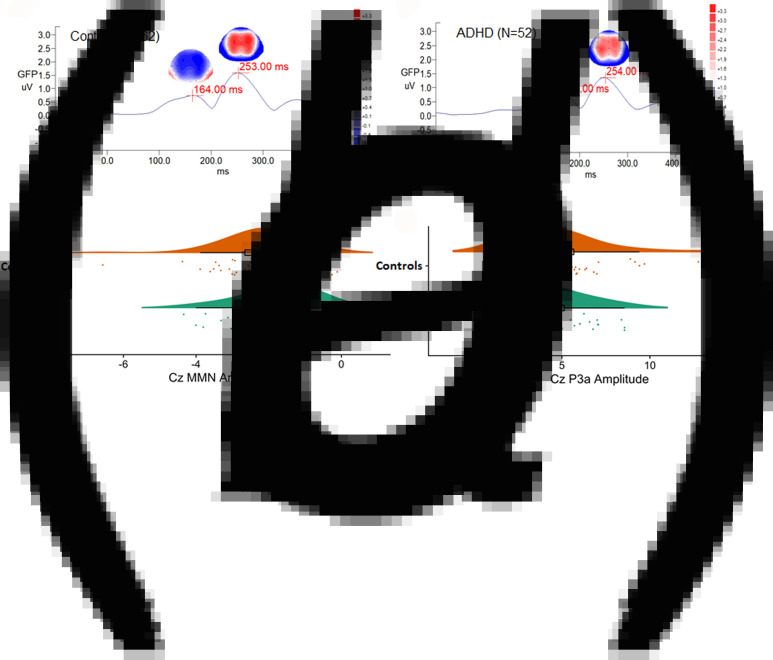
A magnified version of Fig. 1*c* grand average MMN/P3a waveforms, showing scalp topography, and global field power in (*a*) adult controls and (*b*) adults with ADHD. Scalp topography (fronto-central maximum and reversed in polarity over the mastoid sites) indicated the peaks of MMN (in blue, the most negative deflection identified between 90 and 250 ms post-stimulus interval), followed by P3a (in red, the most positive deflection between 210 and 350 ms post-stimulus interval). Rain cloud plots with boxplots of (*c*) MMN and (*d*) P3a Cz amplitudes in both groups were demonstrated.

There was no sex effect on MMN and P3a parameters, while MMN amplitudes decreased with increasing age (*r* = 0.212, *p* = 0.024) (online Supplementary Table S1). For MMN, comparing to matched controls, ADHD adults revealed the trend of less negative values in Cz, indicating a smaller MMN amplitude (*p* = 0.007 for non-parametric Mann–Whitney test, effect size = 0.295). For P3a, there was no significant group difference in any of the P3a indexes between the ADHD and control groups. Figure 1 illustrates the GFP analysis, while the peaks of GFP in Fig. 1*c* reflect MMN/P3a. Figure 2*a* and *b* demonstrate scalp topography with peaks of MMN and P3a revealed fronto-central maximum for the control and ADHD groups, respectively. The distribution of MMN (Fig. 2*c*) and P3a (Fig. 2*d*) amplitudes was visualized by rain cloud plots with boxplots.

With regard to the real-world executive functions (BRIEF), attention performance (CCPT), and decision making (CGT), we found that compared to controls, ADHD adults demonstrated poorer real-world executive functions on all domains assessed by BRIEF (Table 2), and poorer performance in focused attention, impulsivity, and vigilance but no significant differences on decision-making assessed by CGT.

**Table 2.** Executive functions and attention performance assessed by the BRIEF, CCPT, and Cambridge Gambling Task

Mean ± s.d.	ADHD (*N* = 52)	Controls (*N* = 62)	*F*^a^	*p*
*BRIEF*				
Behavior regulation index	31.17 ± 11.87	12.75 ± 8.59	83.78	*<*0.001
Metacognition index	46.75 ± 18.28	18.41 ± 11.40	92.12	*<*0.001
Global executive composite	77.92 ± 28.92	31.15 ± 19.08	96.78	*<*0.001
*CCPT*				
Focused/sustained attention				
Omission errors	2.02 ± 2.35	1.18 ± 1.94	3.82	0.053
Hit RT s.e.	4.91 ± 1.90	4.07 ± 1.18	7.60	0.007
Variability	6.50 ± 4.81	4.92 ± 2.54	4.55	0.035
*Impulsivity*				
Commission errors	16.59 ± 7.41	13.37 ± 7.07	5.53	0.021
Perseverations	0.82 ± 1.40	0.23 ± 0.77	8.34	0.005
*Vigilance*				
Hit reaction time inter-stimulus intervals	0.07 ± 0.03	0.06 ± 0.02	4.41	0.038
Hit s.e. changed by ISIs	0.04 ± 0.11	−0.0002 ± 0.09	4.68	0.033
*Cambridge Gambling task*				
Overall proportion bet	0.54 ± 0.16	0.52 ± 0.14	0.58	0.449
Ascending	0.42 ± 0.19	0.38 ± 0.17	1.15	0.286
Descending	0.67 ± 0.18	0.67 ± 0.17	0.00	0.998
Risk adjustment	1.39 ± 1.03	1.56 ± 1.19	0.62	0.434
Ascending	1.67 ± 1.26	2.08 ± 1.25	3.36	0.070
Descending	1.28 ± 1.13	1.34 ± 1.31	0.05	0.832
Risk-taking	0.58 ± 0.16	0.56 ± 0.14	0.69	0.407
Ascending	0.46 ± 0.20	0.42 ± 0.18	1.23	0.270
Descending	0.71 ± 0.18	0.71 ± 0.17	0.01	0.931

ADHD, attention-deficit hyperactivity disorder; BRIEF, behavior rating inventory of executive function; CCPT, Conners' continuous performance test; s.d., standard deviation.

a Controling for age and sex.

To determine the relationship between MMN (Cz) amplitude and clinical/neurocognitive parameters, multiple linear regression models with the backward elimination procedure were conducted for the ADHD and control group (Table 3). The results showed that MMN amplitude at Cz was significantly associated with sex, inattention symptoms (SNAP-IV), MCI (initiate, WM, planning and organizing, organization of materials and monitor) on the BRIEF, sustained attention on the CCPT, and overall proportion bet on the CGT in the ADHD group (*R*^2^ = 0.30) and risk adjustment and risk-taking on the CGT in the control group (*R*^2^ = 0.21). The results of the forward and stepwise selection generally supported the findings in backward elimination procedures (data not shown). We found that the results of backward selection in the ADHD group were also supported by the Lasso method with five-fold cross-validation, showing that MCI of BRIEF, and Hit RT ISIs of CCPT were predictive of Cz MMN amplitude in the ADHD group (online Supplementary Table S2). Using different selection methods, we found that inattention, MCI, variables of CGT and Hit RT ISIs of CCPT were generally consistently selected by at least two methods of model selection for MMN amplitude in ADHD, while risk-taking and risk adjustment were consistently selected in the model for the control group.

**Table 3.** Models of the link between Cz MMN amplitude and neurocognitive functions in adults with ADHD *v* control subjects using backward selection

Models	ADHD (*N* = 52)		Controls (*N* = 62)	
	Cz MMN amplitude		Cz MMN amplitude	
	*ß*	s.e.	*ß*	s.e.
Sex	−0.55*	0.31	–	–
*SNAP-IV*				
Inattentive	−0.08**	0.03	–	–
*BRIEF*				
Metacognition index	0.03***	0.01	–	–
*CCPT*				
Vigilance				
Hit reaction time ISI	−18.24***	5.71	–	–
Hit s.e. changed by ISI	3.35**	1.52	–	–
*CANTAB*				
Cambridge Gambling task				
Overall proportion bet				
Descending	−1.45*	0.76	–	–
Risk adjustment				
Ascending	–	–	0.22*	0.12
Descending	–	–	−0.49***	0.17
Risk-taking				
Descending	–	–	−4.13****	1.15
*R*^2^	0.30		0.21	

Abbreviations: SNAP-IV, the Chinese version of the Swanson, Nolan, and Pelham, version IV scale*;* BRIEF, behavior rating inventory of executive function; CCPT, Conners' continuous performance test; ISI, inter-stimulus interval; CANTAB, Cambridge neuropsychological test automated battery.

*p *<* 0.1; **p *<* 0.05; ***p *<* 0.01; ****p *<* 0.001.

## Discussion

With attention deficits and executive dysfunctions as the core features of ADHD across the lifespan (Faraone *et al*. 2015; Lin & Gau, 2019), whether the brain response towards novelty detection (MMN) and attention reorientation (P3a) altered in adults with ADHD or not has not been studied before. This work is the first study to examine the MMN and P3a using a passive auditory oddball paradigm and their correlations with ADHD symptoms and neuropsychological functions in drug-naive adults with ADHD. We found that adults with ADHD showed decreased Cz amplitude in MMN, yet no group differences in P3a. Our results further showed that MMN at Cz demonstrated different correlations patterns with clinical and neuropsychological measures across the ADHD and control groups using backward model selections. ADHD-specific patterns of clinical/neurocognitive correlates for MMN at Cz showed that inattention, vigilance, metacognition (WM, organization, planning, and monitoring), and risk adjustment for decision-making were significantly associated with MMN at Cz. However, only risk adjustment and risk-taking were significantly associated with MMN at Cz in controls. Our finding is novel since no such kind of study has been conducted before.

The main finding of smaller MMN amplitude at Cz in ADHD adults than controls is consistent with previous studies (Cheng *et al*. 2016; Rydkjaer *et al*. 2017) and supports our hypothesis of preattentive change detection deficits in adults with ADHD. Due to ADHD core symptoms, patients with ADHD usually could not persistently cooperate well on the cognitive task but tend to make careless mistakes during the tasks. This study, using a passive auditory paradigm that does not require the participants to provide a response and be motivated to cooperate, demonstrated that the ADHD adults showed MMN amplitude, which was significantly reduced at Cz with a small to medium effect size. Our study extends the knowledge of reduced MMN amplitude in ADHD children concluded by Cheng's meta-analysis (Cheng *et al*. 2016) and provides evidence that ADHD adults also demonstrated reduced MMN amplitude. Notably, most studies in Cheng's meta-analysis in ADHD children measured frequency MMN. Our paradigm, measuring duration MMN, consistently showed MMN amplitude reduction in ADHD. Collectively, ADHD individuals may have deficits in preattentive change detection, no matter to frequency deviants or duration deviants, and such deficits may persist into adulthood. In other words, MMN amplitude reduction may serve as a physiological biomarker of ADHD.

Similar to MMN, there was no previous research that focused on P3a in adults with ADHD either. Rydkjaer *et al*. (2017) assessed young adolescents (12 to 17 years of age) using a four-tone auditory oddball task (with deviant stimuli on frequency/duration/combination) and found that P3a amplitudes in ADHD youths were not significantly different from those of healthy controls and first-episode psychosis patients. Our study, using a simpler auditory paradigm in ADHD adults, discovered that there was no significant difference between ADHD and controls in P3a.

Our finding that smaller MMN at Cz was correlated with inattention symptoms and executive dysfunction is of particular interest. Deficits in preattentive change detection measures by MMN were related to higher inattentive symptoms but not hyperactivity or impulsivity. To our knowledge, there was only one study with a smaller sample size focusing on the relationship between MMN and ADHD-symptom severity (Yamamuro *et al*. 2016), which reported an association between MMN amplitude at Pz and attention deficits indeed corresponds to our finding at Cz. In contrast, their finding of an association between MMN amplitude and hyperactivity/impulsivity was not shown in our data. This discrepancy is possibly due to the severity of hyperactivity/impulsivity often reduced from childhood to adulthood while the inattention persisted into adulthood (Faraone *et al*. 2015; Lin *et al*. 2016; Lin & Gau, 2019). Such specific correlations in ADHD adults further strengthen the notion that MMN is not only a physiological marker of ADHD but also correlates well with the overall attention ability. Intriguingly, MMN reflects a preattentive involuntary change detection, how a marker that is attention independent related with inattention symptoms warrants further investigation. Perhaps the inattention symptoms of ADHD in daily life are primarily explained by deficits in change detection in the very early stage of information processing that they cannot either aware of it. In this way, they made careless mistakes, but they could not find their errors and frequently denied their mistakes unless being pointed out.

The correlations between MMN reduction and executive dysfunction in daily life (i.e. initiation, WM, organization, planning, and monitoring) implied that MMN at Cz differentiated ADHD adults from adult controls not only on the dimensions of attention but executive functions as well. MMN represents the preattentive process of auditory discrimination and is associated with the function of auditory memory and involuntary attention shifting (Javitt *et al*. 1995; Naatanen *et al*. 2007; Naatanen & Michie, 1979). Change detection is an essential part of several executive functions. A previous study used the MMN paradigm to investigate temporal processing elicited by time-based stimulus features to a number of cognitive functions in a non-clinical sample (Foster *et al*. 2013). They found that executive functions (i.e. planning and conditional inhibition, but not set-shifting) uniquely predicted variance in temporal processing (Foster *et al*. 2013). Another study that investigated the relationship between deficits of tone duration MMN and executive functions in patients with schizophrenia also demonstrated a significant correlation between low MMN amplitude and poor performances of executive functions assessed by the Wisconsin Card Sorting Test, Stroop Test, and Trail Making Test (Toyomaki *et al*. 2008). Combined with our findings within ADHD and control adults, lower MMN amplitude may have neurocognitive implications across non-clinical samples and different clinical groups, particularly for executive functions. How preattentive change detection contributes to executive dysfunction is worth further research.

When using model selection to pick up significant correlates of MMN at Cz, we found that correlates predicting MMN in ADHD was remarkably distinct from those in controls. In the control group, only risk adjustment and risk-taking parameters of the CGT were associated with MMN amplitude at Cz. In contrast, in ADHD adults, sex, inattention, metacognition, vigilance on the CCPT, as well as overall proportion bit of the CGT remained in the model for predicting MMN amplitude at Cz. To date, there has been no study focusing on the effect of risk-taking behavior (measured by gambling test) or vigilance (captured by the CCPT) on MMN amplitude within the ADHD population. Vigilance refers to the brain alertness for the objective stimuli, including the concentration of attention and the capability to respond to emergencies. Evidence has shown that the MMN amplitude reduces significantly when unattended vigilance on the modified Mackworth Clock Test is going down (Chang *et al*. 2016). Besides, EEG has been proposed to be used for monitoring individual vigilance changes over time (Kim, Kim, & Im, 2017). Our findings provide further evidence to support the relationship between the MMN amplitude and vigilance modulation in ADHD adults and warrant research attention.

On the other hand, recent studies suggest that predictive coding is a useful conceptual framework for understanding MMN generating mechanisms (McCleery *et al*. 2019; Tada *et al*. 2019; Wacongne, 2016; Wacongne, Changeux, & Dehaene, 2012). Predictive coding is a hierarchical information processing model that posits interactions between lower-order perceptual signals and higher-order cognitive processes in a dynamic and iterative manner to generate predictions about the environment and compare incoming stimuli with these predictions (Chang *et al*. 2016; McCleery *et al*. 2019; Nazimek, Hunter, & Woodruff, 2012). According to this model, neural responses to stimuli that match predictions are suppressed, whereas stimuli that are unexpected, violating these predictions, trigger a mismatch “prediction error” signal (Garrido, Kilner, Stephan, & Friston, 2009). Similar to the process of risk adjustment or risk-taking in the gambling test (CGT), the prediction error signals that do the updating of expectations is required to accommodate the discrepant stimuli. Such a notion was supported by our data that MMN amplitude was related to risk adjustment and risk-taking in adult controls but not in adults with ADHD, which possibly implied that MMN deficits might reflect more on the inattention, executive dysfunction, and vigilance impairment than in risk adjustment/taking in ADHD, a differential associated pattern from the controls.

Several features constitute the strengths of this study, including the first study of MMN and P3a using a passive oddball paradigm in adult ADHD, a larger sample than any previous ERP ADHD studies, drug-naive, and comprehensive assessments. Selection bias is the major methodological limitation of this study, including male predominance, recruitment only from one medical center, and no comorbid psychiatric conditions and psychotropic exposures. Hence, the generalizability of our results is questionable. Future studies will need to assess whether several of our novel findings can be validated by other populations and generalized to the whole ADHD population. Second, due to the restriction of the sample to a more homogenous group who could finish all the neurocognitive tasks and ERP in this study, the generalization of the findings to other populations who could not complete all the measures may be questionable. Third, whether the different passive auditory paradigm (MMN and P3a) in patients with ADHD reflects the underlying neuropathology of ADHD or the consequences of a compensatory neurodevelopment process cannot be determined in this cross-sectional study.

This study provides the first data that the passive auditory paradigm (MMN) is highly associated with inattention symptoms, real-world executive dysfunctions (i.e. initiation, WM, organization, monitoring, planning, problem-solving), vigilance on the CCPT, and risk adjustment on gambling task in adults with ADHD compared to adult controls of comparable age, education level, employment status, and IQ. Our findings of decreased MMN amplitude as well as differential correlates for the preattentive process of novelty detection in ADHD adults from those in controls provide strong evidence to support the validity of adult ADHD based on the electrophysiological marker for adult ADHD (i.e. Cz amplitude of MMN). It is warranted to investigate other neurophysiological markers theoretically associated with ADHD neuropathology, and to collect longitudinal data to shed light on the developmental trajectories in ADHD.

## Supplementary material

For supplementary material accompanying this paper visit https://doi.org/10.1017/S0033291720005516. click here to view supplementary material
