# Supplementary material for: Mismatch negativity and P3a in drug-naive adults with attention-deficit hyperactivity disorder
Source: Psychol Med. 2021 Mar 12;52(15):3360–70. doi: 10.1017/S0033291720005516 (PMC9772912; doi:10.1017/S0033291720005516)
Supplement: Supplementary file 1 [file S0033291720005516sup001.docx]

**Mismatch negativity and P3a in drug-naïve adults with attention-deficit hyperactivity disorder**

Ming H. Hsieh, MD, PhD ^1^, Yi-Ling Chien, MD, PhD ^1^, Susan Shur-Fen Gau, MD, PhD*^1,2^

^1^ Department of Psychiatry, National Taiwan University Hospital and College of Medicine, Taipei, Taiwan

^2^ Graduate Institute of Brain and Mind Sciences, and Graduate Institute of Clinical Medicine, College of Medicine, National Taiwan University, Taipei, Taiwan

**Supplementary Methods, Tables (S1, S2) and Figures**

**Behavioral Measures**

***The Chinese version of the Swanson, Nolan, and Pelham, version IV scale (SNAP-IV).*** The participants reported their ADHD-related symptoms on the SNAP-IV (Swanson *et al.*, 2001), which is a 26-item scale rating on a 4-point Likert scale with a score of 0 for "not at all," to 3 for "very much." There are 18 items parallel to the core symptoms of DSM-IV ADHD (Items 1-9 for inattention symptoms; Items 10-18 for hyperactivity/impulsivity symptoms), and 8 items (Items 19-26) based on the DSM-IV of oppositional defiant disorder symptoms. The psychometric properties and norm of the parent (Gau *et al.*, 2008a) and teacher reports (Gau *et al.*, 2009) on the Chinese SNAP IV have been established, showing the same 3-factor structure as its English version (Gau *et al.*, 2008a). The SNAP-IV has been used for adults to report their ADHD-related symptoms (Yang *et al.*, 2013). The Chinese SNAP-IV has been widely used in clinical and research settings in Taiwan regarding treatment studies (Chou *et al.*, 2009, Gau *et al.*, 2008b, Gau & Shang, 2010), epidemiological studies (Chen *et al.*, 2015, Chen *et al.*, 2017, Chen *et al.*, 2019, Gau *et al.*, 2008a, Gau *et al.*, 2009, Tsai *et al.*, 2017), and clinical studies (Chen *et al.*, 2015, Chiang *et al.*, 2015, Chiang *et al.*, 2016, Chiang & Gau, 2016, Chiang *et al.*, 2019, Chiang & Gau, 2008, Chien *et al.*, 2014, Liu *et al.*, 2011, Shang *et al.*, 2015, Shang *et al.*, 2013, Tsai *et al.*, 2013, Yang *et al.*, 2013). The participants only reported their ADHD symptoms, i.e., inattention and hyperactivity-impulsivity, on the first 18 items of the SNAP-IV. In this study, we only include the first 18 items of the SNAP-IV in the clinical report form.

***The Behavior Rating Inventory of Executive Function (BRIEF) (Gioia et al., 2000).*** The BRIEF is an 86-item questionnaire reported by adults aged 18 and older. The BRIEF is developed to evaluate daily-life or real-world behaviors related to [executive function](https://en.wikipedia.org/wiki/Executive_function)s for adults to report on themselves (Baron, 2000). The BRIEF measures are comprised of eight clinical subscales which are collapsed into two broad indices: (1) Behavioral Regulation Index (BRI); Inhibit, Shift and Emotional Control; and (2) Metacognition Index (MCI): Initiate, Working Memory, Planning and Organizing, Organization of Materials, and Monitor; as well as an overall index (Global Executive Composite, GEC). The items are rated on a frequency level (1 for "Never," 2 for "Sometimes," and 3 for "Often"). The BRIEF has been reported to have excellent psychometric properties, including high test-retest and interrater reliability, as well as face and predictive validity (Baron, 2000). The Chinese version of the BRIEF has been used in epidemiological (Tsai *et al.*, 2019) and clinical (Goto *et al.*, 2017) research.

**Neurocognitive tasks**

***Conner's Continuous Performance Test (CCPT)***.

The CCPT is a 14-minute, widely-used computerized task for individuals aged 6 and up (Conners & Staff, 2000). It is a non-X type CPT test of go/no-go paradigm, which requires subjects to tap on the spacebar when any character except *X* is shown on the screen (Conners & Staff, 2000). There are six blocks in CCPT, with three sub-blocks each containing 20-letter presentations, resulting in 360 trials in total. The 360 trials, composed of 10% no-go targets, were presented with six blocks and three sub-blocks (20 trials in each sub-block). The sub-blocks differ in Inter-Stimulus Intervals (ISIs) of 1, 2, and 4 seconds, and the sequence of ISI conditions is presented randomly. Seven indexes are presented in this study to address the three attention profiles proposed by Egeland and Kovalik-Gran (Egeland & Kovalik-Gran, 2010): (1) focused attention: omission errors, reaction time (RT) variability, Hit RT standard errors (SE); (2) cognitive impulsivity: commission errors, perseverations (defined as the responses occurring less than 100 ms following the stimuli that are too quick for a respondent to react); and (3) vigilance: Hit RT and Hit RT SE changed across different ISIs (i.e., slower and more variable RT with more extended ISI changes).

**Cambridge Gambling Test (CGT)** CGT is one of the tasks of the Cambridge Neuropsychological Test Automated Battery (CANTAB, Cambridge Cognition Ltd), which is a set of computerized tests to examine nonverbal neuropsychological functions. CGT is designed to assess decision-making capabilities (Rogers *et al.*, 1999), while the participant is presented with ten boxes, colored either red or blue, and appeared in varying ratios (6:4, 7:3, 8:2, 9:1) of red to blue. They are informed that a yellow token is hidden in one of the boxes. At the bottom of the screen are two response boxes, one for each color. The participant must use such information to guess whether the token is hidden under a red or blue box. If the participant had located the hidden token correctly, then the points they wagered were added to their total score. If they had made the wrong decision, however, then that the same amount was subtracted from their total. Participants were always able to see their point total on the screen. They were able to select their bets from a list of five options calculated by the computer, with the amounts corresponding to 5%, 25%, 50%, 75%, and 95% of their current point total. These bet amounts were presented either in ascending or descending order during CGT administration (ascending and descending conditions). Participants were required to choose a wager from any of these possible amounts within 2 sec. If they failed to do so, then the last bet was automatically set by the computer. Their bets were presented together with a sound, with low-pitched tones indicating low bets, and high-pitched tones indicating high bets. This work shows six indexes: (1) Overall bet proportion: both the ascending and the descending conditions, (2) Risk adjustment: the mean risk-taking score (points) for each box ratio for both the ascending and the descending conditions where points to gamble differ relative to box ratio, and (3) Risk-taking: the total difference between risk-taking scores (points gambled) in the ascending and descending conditions (delay aversion).

**Auditory event-related potentials (ERP) method**

**ERP recording environment**

We followed the standard protocols of the passive auditory oddball paradigm (Duncan *et al.*, 2009, Light *et al.*, 2010) at our lab, which had been used in our previous research (Hsieh *et al.*, 2019, Hsieh *et al.*, 2012, Huang *et al.*, 2018, Lin *et al.*, 2014, Lin *et al.*, 2012). Audiometry testing was performed before ERP recording to exclude subjects who could not detect 40-dB sound pressure level tones at 500, 1000, and 6000Hz presented to either ear. Auditory stimuli were presented binaurally by foam insert headphones. The stimuli were generated by, and data was recorded by Neuroscan STIM and ACQUIRE system [Neuroscan, El Paso, Tex.]. Electrodes were used at 32 recording sites utilizing Neuroscan QuikCaps (According to QuikCap website: All electrodes were placed according to the International 10-20 electrode placement standard). Electrodes placed at the tip of the nose and Fpz served as the reference and ground, respectively. Four additional electrodes placed above and below the left eye and at the outer canthi of both eyes are used for monitoring blinks and eye movements. All impedances would be below 5 kΩ. Signals were digitized at a rate of 1 kHz with system acquisition filter settings of 0.5 to 100 Hz, with no 60Hz notch filter.

**MMN/P3a session**

ERP data were collected while participants viewed a benign cartoon film while the cartoon soundtrack was turned off and replaced by the experimental tones. To minimize eye movement and muscle artifact during the recording, the cartoon film is presented at eye level on a 19-inch LCD monitor screen. A duration-deviant auditory oddball paradigm was employed, as standard (p = 0.90, 50-ms duration) and deviant (p = 0.10, 100-ms duration) tones were presented in pseudorandom order with at least 2 standards are presented before each deviant. All stimuli were 1,000 Hz and 80dB with 1 msec rise-fall time and presented at a fixed 500msec onset-to-onset asynchrony. During the passive auditory paradigm, subjects were closely observed through a video monitor. They would be monitored visually and by EEG for signs of sleep or slow-wave activity, which, if present, prompted the experimenter to speak briefly with the subject. In addition, online ERP averages to standard and deviant tones were also collected to monitored signal quality and the number of sweeps free of gross artifacts (defined as ±100 μV across the 100~500 msec following stimuli). EEG acquisition was terminated when a minimum of 225 artifact-free deviant trials were collected, while the whole session took over approximately a 30min period.

**Offline data processing**

Offline data processing was performed with automated procedures utilizing Neuroscan Scan 4.5 software blind to clinical group in the following ways: (1) continuous recordings were mathematically corrected for eye movement artifact using established methods (Semlitsch *et al.*, 1986); (2) digital bandpass filtering within 1 to 40 Hz; (3) continuous data were divided into epochs relative to the onset of stimuli within −100 to 500 milliseconds range; (4) automatic removal of artifacts by excluding the epochs containing amplitude differences of ±50 μV in frontal recording sites; (5) construction of the MMN/P3a waves by subtracting the averaged standard ERP from each of the averaged deviant ERP; (6) the resultant MMN subtraction waveforms were lowpass filtered at 20 Hz (phase shift and 24dB/octave roll-off) to remove residual high-frequency artifact; (7) individual scoring of maximum MMN amplitudes between 90 and 250 msec and of maximum P3a amplitude between 210 and 350 msec. MMN and P3a amplitudes were assessed from the midline electrodes Cz for analysis (Duncan *et al.*, 2009, Rydkjaer *et al.*, 2017).

**Supplementary Table S1.** Pearson's correlations between age and MMN/P3a parameters.


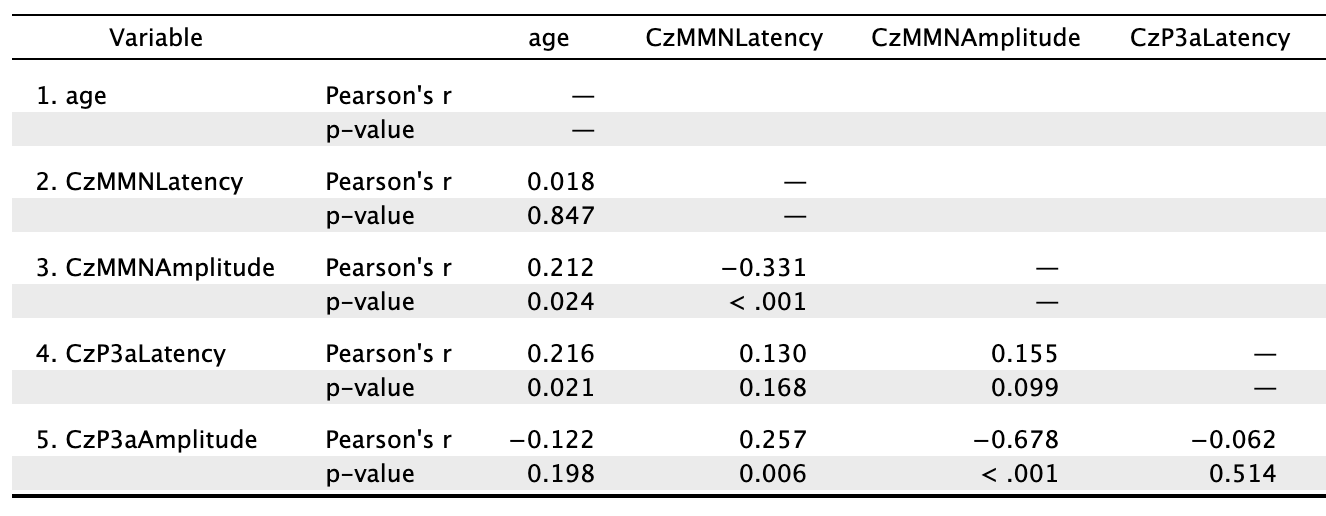


**Supplementary Table S2.** Lasso method for MMN amplitude in ADHD

| **Parameter** | **DF** | **Estimate** | **Cross-validation estimate** | | | | |
| --- | --- | --- | --- | --- | --- | --- | --- |
|  |  |  | 1 | 2 | 3 | 4 | 5 |
| Intercept | 1 | -2.152 | 0.0383 | -0.0440 | -0.123 | -0.0177 | 0.0996 |
| Metacognition Index | 1 | 0.006 | 0.1990 | 0.5111 | 0.680 | 0.4398 | 0.6808 |
| Hit reaction time:  inter-stimulus intervals | 1 | -0.817 | -0.6364 | -0.5181 | -0.212 | -0.1938 | -0.3992 |
| Cambridge gambling task: risk adjustment | 1 | 0.133 | 0.0833 | 0.0622 | 0.416 | 0.3398 | -0.0211 |

**Supplementary Figure**

Associations between MMN and behavioral parameters in the ADHD and control groups, corresponding to Table 3.

| **ADHD:** Cz MMN amplitude vs. Inattentive  *r*=0.117, *p*=0.409 | **ADHD:** Cz MMN amplitude vs. Overall proportion bet (Descending)  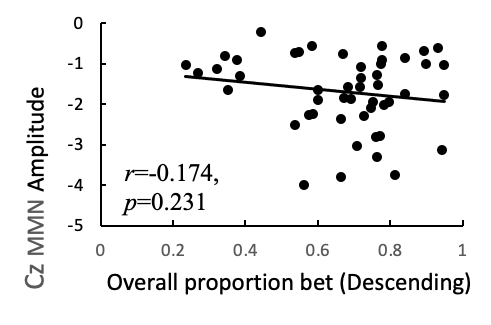 |
| --- | --- |
| **ADHD:** Cz MMN amplitude vs. Metacognition Index  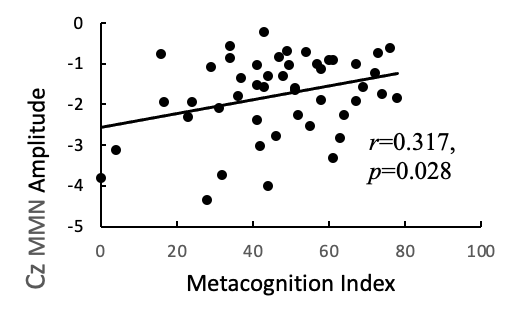 | **Controls:** Cz MMN amplitude vs.  Risk adjustment (Ascending)  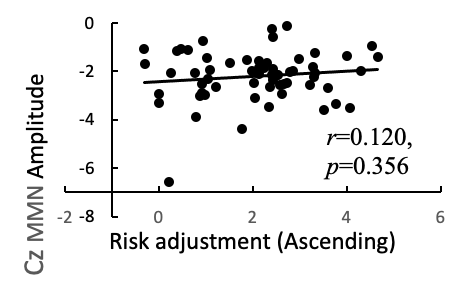 |
| **ADHD:** Cz MMN amplitude vs. Hit reaction time ISI  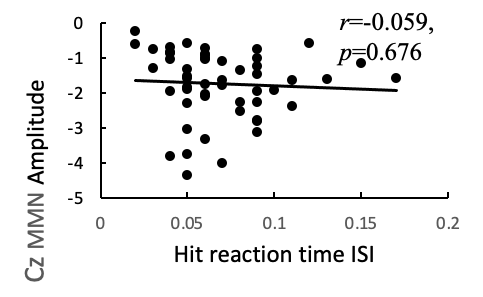 | **Controls:** Cz MMN amplitude vs.  Risk adjustment (Descending)  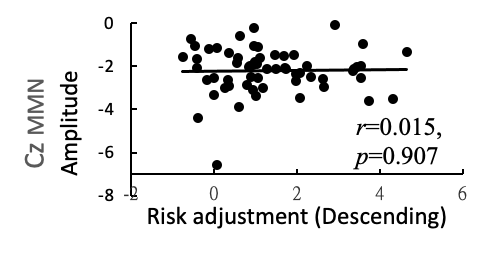 |
| **ADHD:** Cz MMN amplitude vs. Hit standard error changed by ISI  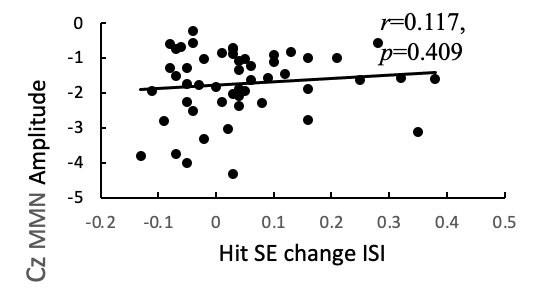 | **Controls:** Cz MMN amplitude vs.  Risk taking (Descending)  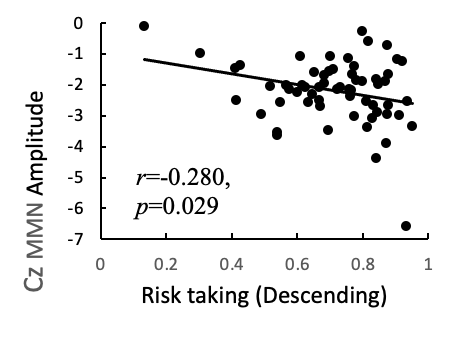 |

**References**

Baron, I. S. (2000). Behavior rating inventory of executive function. *Child Neuropsychology*, 6(3), 235-238. doi: 10.1076/chin.6.3.235.3152.

Chen, Y. L., Chen, S. H. & Gau, S. S. (2015). ADHD and autistic traits, family function, parenting style, and social adjustment for Internet addiction among children and adolescents in Taiwan: A longitudinal study. *Research in Developmental Disabilities*, 39, 20-31. doi: 10.1016/j.ridd.2014.12.025.

Chen, Y. L., Shen, L. J. & Gau, S. S. (2017). The mandarin version of the Kiddie-Schedule for Affective Disorders and Schizophrenia-Epidemiological version for DSM-5 - A psychometric study. *Journal of Formosan Medical Association*, 116(9), 671-678. doi: 10.1016/j.jfma.2017.06.013.

Chen, Y. Y., Chen, Y. L. & Gau, S. S. (2019). Attention-deficit hyperactivity disorder and suicidality: The mediating effects of psychiatric comorbidities and family function. *Journal of Affective Disorders*, 242, 96-104. doi: 10.1016/j.jad.2018.08.023.

Chiang, H. L., Chen, Y. J., Lo, Y. C., Tseng, W. Y. & Gau, S. S. (2015). Altered white matter tract property related to impaired focused attention, sustained attention, cognitive impulsivity and vigilance in attention-deficit/ hyperactivity disorder. *Journal of Psychiatry and Neuroscience*, 40(5), 325-335. doi: 10.1503/jpn.140106.

Chiang, H. L., Chen, Y. J., Shang, C. Y., Tseng, W. Y. & Gau, S. S. (2016). Different neural substrates for executive functions in youths with ADHD: a diffusion spectrum imaging tractography study. *Psychological Medicine*, 46(6), 1225-1238. doi: 10.1017/S0033291715002767.

Chiang, H. L. & Gau, S. S. (2016). Comorbid psychiatric conditions as mediators to predict later social adjustment in youths with autism spectrum disorder. *Journal of Child Psychology and Psychiatry and Allied Disciplines*, 57(1), 103-111. doi: 10.1111/jcpp.12450.

Chiang, H. L., Hsu, Y. C., Shang, C. Y., Tseng, W. I. & Gau, S. S. (2019). White matter endophenotype candidates for ADHD: a diffusion imaging tractography study with sibling design. *Psychological Medicine*, 1-11. doi: 10.1017/S0033291719001120.

Chiang, M. & Gau, S. S. (2008). Validation of attention-deficit-hyperactivity disorder subtypes among Taiwanese children using neuropsychological functioning. *Australian and New Zealand Journal of Psychiatry*, 42(6), 526-535. doi: 10.1080/00048670802050603.

Chien, Y.-L., Gau, S. S.-F., Chiu, Y.-N., Tsai, W.-C., Shang, C.-Y. & Wu, Y.-Y. (2014). Impaired sustained attention, focused attention, and vigilance in youths with autistic disorder and Asperger's disorder. *Research in Autism Spectrum Disorders*, 8(7), 881-889. doi: https://doi.org/10.1016/j.rasd.2014.04.006.

Chou, W. J., Chou, M. C., Tzang, R. F., Hsu, Y. C., Gau, S. S., Chen, S. J., . . . Cheng, H. (2009). Better efficacy for the osmotic release oral system methylphenidate among poor adherents to immediate-release methylphenidate in the three ADHD subtypes. *Psychiatry and Clinical Neurosciences*, 63(2), 167-175. doi: 10.1111/j.1440-1819.2009.01937.x.

Conners, C. K. & Staff, M. (2000). *Conners’ Continuous Performance Test II: Computer Program for Windows Technical Guide and Software Manual*. Mutli-Health Systems.: North Tonwanda, NY.

Duncan, C. C., Barry, R. J., Connolly, J. F., Fischer, C., Michie, P. T., Naatanen, R., . . . Van Petten, C. (2009). Event-related potentials in clinical research: guidelines for eliciting, recording, and quantifying mismatch negativity, P300, and N400. *Clin Neurophysiol*, 120(11), 1883-1908. doi: 10.1016/j.clinph.2009.07.045.

Egeland, J. & Kovalik-Gran, I. (2010). Validity of the factor structure of Conners’ CPT. *Journal of Attention Disorders*, 13(4), 347-357. doi: 10.1177/1087054709332477.

Gau, S. S.-F., Shang, C.-Y., Liu, S.-K., Lin, C.-H., Swanson, J. M., Liu, Y.-C. & Tu, C.-L. (2008a). Psychometric properties of the Chinese version of the Swanson, Nolan, and Pelham, version IV scale – parent form. *International Journal of Methods in Psychiatric Research*, 17(1), 35-44. doi: 10.1002/mpr.237.

Gau, S. S., Chen, S. J., Chou, W. J., Cheng, H., Tang, C. S., Chang, H. L., . . . Huang, Y. S. (2008b). National survey of adherence, efficacy, and side effects of methylphenidate in children with attention-deficit/hyperactivity disorder in Taiwan. *Journal of Clinical Psychiatry*, 69(1), 131-140. doi: 10.4088/jcp.v69n0118.

Gau, S. S., Lin, C. H., Hu, F. C., Shang, C. Y., Swanson, J. M., Liu, Y. C. & Liu, S. K. (2009). Psychometric properties of the Chinese version of the Swanson, Nolan, and Pelham, Version IV Scale-Teacher Form. *Journal of Pediatric Psychology*, 34(8), 850-861. doi: 10.1093/jpepsy/jsn133.

Gau, S. S. & Shang, C. Y. (2010). Improvement of executive functions in boys with attention deficit hyperactivity disorder: an open-label follow-up study with once-daily atomoxetine. *International Journal of Neuropsychopharmacology*, 13(2), 243-56. doi: 10.1017/S1461145709990836.

Gioia, G. A., Isquith, P. K., Guy, S. & Kenworthy, L. (2000). *BRIEF: Behavior Rating Inventory of Executive Function*. Psychological Assessment Resources: Odessa, FL.

Goto, T., Hirata, Y., Takita, Y., Trzepacz, P. T., Allen, A. J., Song, D. H., . . . Takahashi, M. (2017). Efficacy and safety of atomoxetine hydrochloride in Asian adults with ADHD: A multinational 10-week randomized double-blind placebo-controlled Asian study. *Journal of Affective Disorders*, 21(2), 100-109. doi: 10.1177/1087054713510352.

Hsieh, M. H., Lin, Y.-T., Chien, Y.-L., Hwang, T.-J., Hwu, H.-G., Liu, C.-M. & Liu, C.-C. (2019). Auditory event-related potentials in antipsychotic-free subjects with ultra-high-risk state and first-episode psychosis. *Frontiers in Psychiatry*, 10(223). doi: 10.3389/fpsyt.2019.00223.

Hsieh, M. H., Shan, J. C., Huang, W. L., Cheng, W. C., Chiu, M. J., Jaw, F. S., . . . Liu, C. C. (2012). Auditory event-related potential of subjects with suspected pre-psychotic state and first-episode psychosis. *Schizophrenia Research*, 140, 243-249. doi: 10.1016/j.schres.2012.06.021.

Huang, W. L., Liu, C. Y., Liu, C. M., Liu, H. M., Yang, C. Y., Hwang, T. J., . . . Hwu, H. G. (2018). Association between mismatch negativity and voxel-based brain volume in schizophrenia. *Clinical Neurophysiology*, 129(9), 1899-1906. doi: 10.1016/j.clinph.2018.06.018.

Light, G. A., Williams, L. E., Minow, F., Sprock, J., Rissling, A., Sharp, R., . . . Braff, D. L. (2010). Electroencephalography (EEG) and event-related potentials (ERPs) with human participants. *Current Protocols in Neuroscience*, 52(1), 6.25.1-6.25.24. doi: 10.1002/0471142301.ns0625s52.

Lin, Y. T., Hsieh, M. H., Liu, C. C., Hwang, T. J., Chien, Y. L., Hwu, H. G. & Liu, C. M. (2014). A recently-discovered NMDA receptor gene, GRIN3B, is associated with duration mismatch negativity. *Psychiatry Research*, 218(3), 356-358. doi: 10.1016/j.psychres.2014.04.032.

Lin, Y. T., Liu, C. M., Chiu, M. J., Liu, C. C., Chien, Y. L., Hwang, T. J., . . . Hwu, H. G. (2012). Differentiation of schizophrenia patients from healthy subjects by mismatch negativity and neuropsychological tests. *PLoS One*, 7(4), e34454. doi: 10.1371/journal.pone.0034454.

Liu, S. T., Tsai, F. J., Lee, W. T., Lee, C. M., Fan, P. C., Lin, W. S., . . . Gau, S. S. (2011). Attentional processes and ADHD-related symptoms in pediatric patients with epilepsy. *Epilepsy Research*, 93(1), 53-65. doi: 10.1016/j.eplepsyres.2010.10.012.

Rogers, R. D., Owen, A. M., Middleton, H. C., Williams, E. J., Pickard, J. D., Sahakian, B. J. & Robbins, T. W. (1999). Choosing between small, likely rewards and large, unlikely rewards activates inferior and orbital prefrontal cortex. *The Journal of Neuroscience*, 19(20), 9029-9038. doi: 10.1523/jneurosci.19-20-09029.1999.

Rydkjaer, J., Mollegaard Jepsen, J. R., Pagsberg, A. K., Fagerlund, B., Glenthoj, B. Y. & Oranje, B. (2017). Mismatch negativity and P3a amplitude in young adolescents with first-episode psychosis: a comparison with ADHD. *Psychological Medicine*, 47(2), 377-388. doi: 10.1017/S0033291716002518.

Semlitsch, H. V., Anderer, P., Schuster, P. & Presslich, O. (1986). A solution for reliable and valid reduction of ocular artifacts, applied to the P300 ERP. *Psychophysiology*, 23(6), 695-703. doi: 10.1111/j.1469-8986.1986.tb00696.x.

Shang, C. Y., Pan, Y. L., Lin, H. Y., Huang, L. W. & Gau, S. S. (2015). An open-label, randomized trial of methylphenidate and atomoxetine treatment in children with attention-deficit/hyperactivity disorder. *Journal of Child and Adolescent Psychopharmacology*, 25(7), 566-573. doi: 10.1089/cap.2015.0035.

Shang, C. Y., Wu, Y. H., Gau, S. S. & Tseng, W. Y. (2013). Disturbed microstructural integrity of the frontostriatal fiber pathways and executive dysfunction in children with attention deficit hyperactivity disorder. *Psychological Medicine*, 43(5), 1093-1107. doi: 10.1017/S0033291712001869.

Swanson, J. M., Kraemer, H. C., Hinshaw, S. P., Arnold, L. E., Conners, C. K., Abikoff, H. B., . . . Wu, M. I. N. (2001). Clinical relevance of the primary findings of the MTA: Success rates based on severity of ADHD and ODD symptoms at the end of treatment. *Journal of the American Academy of Child & Adolescent Psychiatry*, 40(2), 168-179. doi: 10.1097/00004583-200102000-00011.

Tsai, C. J., Chen, Y. L., Lin, H. Y. & Gau, S. S. (2017). One-year trajectory analysis for ADHD symptoms and its associated factors in community-based children and adolescents in Taiwan. *Child and Adolescent Psychiatry and Mental Health*, 11, 28. doi: 10.1186/s13034-017-0165-4.

Tsai, F. J., Liu, S. T., Lee, C. M., Lee, W. T., Fan, P. C., Lin, W. S., . . . Gau, S. S. (2013). ADHD-related symptoms, emotional/behavioral problems, and physical conditions in Taiwanese children with epilepsy. *Journal of Formosan Medical Association*, 112(7), 396-405. doi: 10.1016/j.jfma.2011.08.022.

Tsai, T. H., Chen, Y. L. & Gau, S. S. (2019). Relationships between autistic traits, insufficient sleep, and real-world executive functions in children: a mediation analysis of a national epidemiological survey. *Psychological Medicine*, 1-8. doi: 10.1017/S0033291719003271.

Yang, H. N., Tai, Y. M., Yang, L. K. & Gau, S. S. (2013). Prediction of childhood ADHD symptoms to quality of life in young adults: adult ADHD and anxiety/depression as mediators. *Research in Developmental Disabilities*, 34(10), 3168-3181. doi: 10.1016/j.ridd.2013.06.011.
